# Upadacitinib successfully treats Hailey-Hailey disease: a cases report and literature review

**DOI:** 10.3389/fimmu.2025.1676459

**Published:** 2026-01-14

**Authors:** Zhuochen Wu, Qing Zhu, Xu Yang, Xiaoyu Xie, Guoqiang Zhang

**Affiliations:** 1 Department of Dermatology, The First Hospital of Hebei Medical University, Shijiazhuang, Hebei, China; 2 Subcenter of National Clinical Research Center for Skin and Immune Diseases, Shijiazhuang, Hebei, China; 3 Hebei Technical Innovation Center of Dermatology and Medical Cosmetology Technology, Shijiazhuang, Hebei, China

**Keywords:** Hailey-Hailey disease, Janus kinase inhibitors, novel therapy, treatment, upadacitinib

## Abstract

Hailey-Hailey disease (HHD) is a rare autosomal dominantly inherited skin disorder first described by brothers Howard and Hugh Hailey in 1939. This article reports a case of refractory Hailey-Hailey disease, which was treated with upadacitinib with remarkable efficacy after the unsatisfactory results of traditional treatment regimens. The patient was a 38-year-old male with a 2-year history of the disease. The rash improved significantly at 3 weeks of oral treatment with upadacitinib 15 mg/day and continued to improve at 12 weeks of follow-up without serious adverse effects. Together with the literature review, this study aims to investigate the efficacy and safety of the JAK inhibitor upadacitinib as a novel therapeutic regimen for Hailey-Hailey disease, and to provide a reference for the clinical treatment of refractory cases.

## Introduction

Hailey-Hailey disease manifests as recurrent erythema, blisters and vesicles in the folds of the axilla and groin, often accompanied by itching and pain, and prone to secondary infections, which seriously affects the quality of life (1). Current treatments include local or systemic use of antimicrobials, glucocorticoids, retinoids, and physical therapy (2, 3), but some patients are ineffective on conventional treatments and have recurrent disease. upadacitinib, a selective JAK1 inhibitor, has been approved for use in autoimmune diseases such as psoriatic arthritis and atopic dermatitis (4, 5), where it acts by inhibiting inflammatory signaling pathways. Recent studies have suggested that Janus kinase inhibitors have some therapeutic potential for blistering skin diseases, but their use in Hailey-Hailey disease has been less reported. In this article, we discuss the efficacy and safety of upadacitinib in the treatment of this disease through a refractory case, combined with a review of the literature, to provide a clinical reference.

## Case report

**Figure 1:**
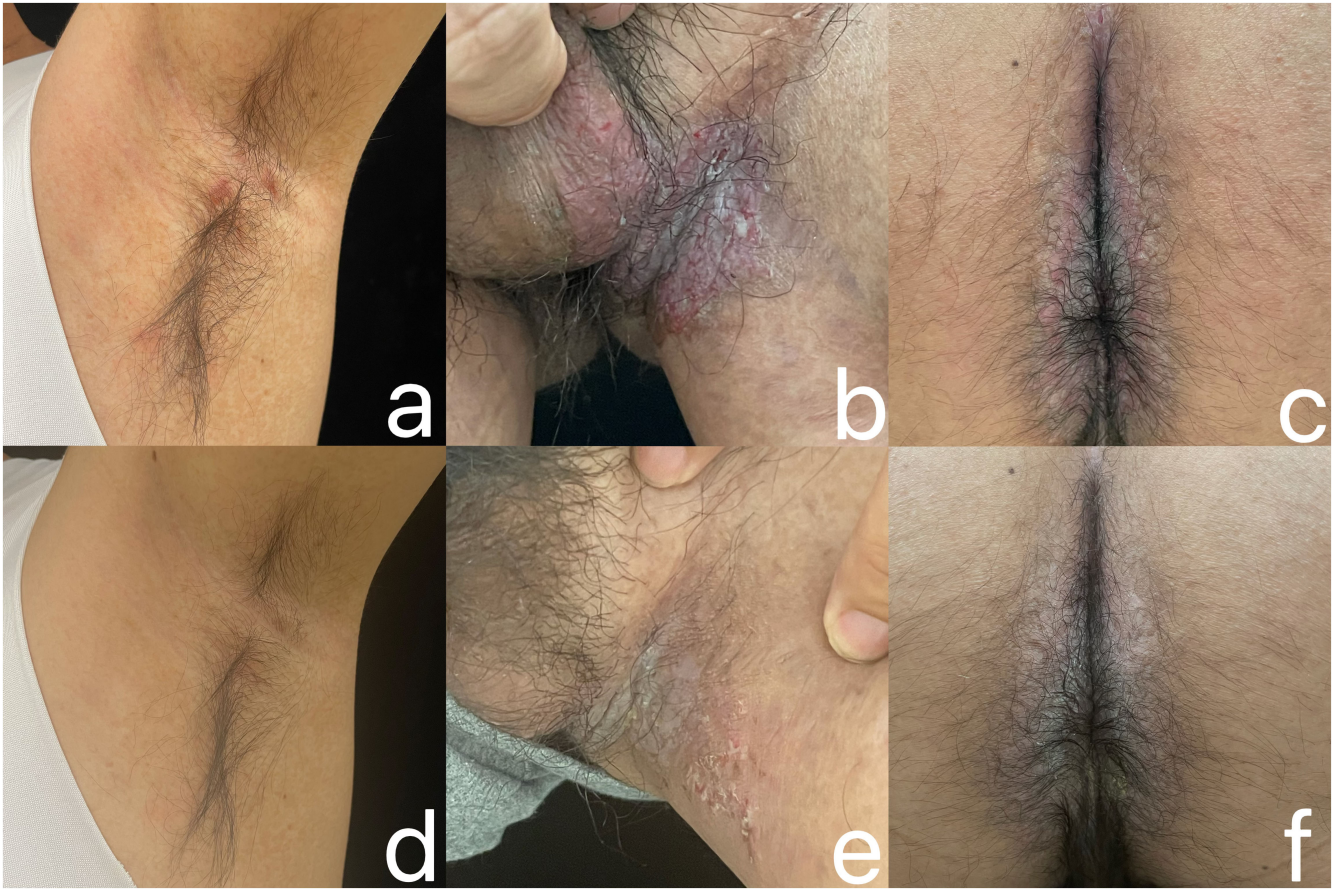
Before treatment, erythema, erosion, and exudation were observed in the left axilla **(a)**, left groin **(b)**, and perianal area **(c)** of the patient in case 2. After 12 weeks of treatment with upadacitinib 15 mg per day, the erythema, erosion, and exudation in the patient’s left axilla **(d)**, left groin **(e)**, and perianal area **(f)** all improved significantly.

**Figure 2:**
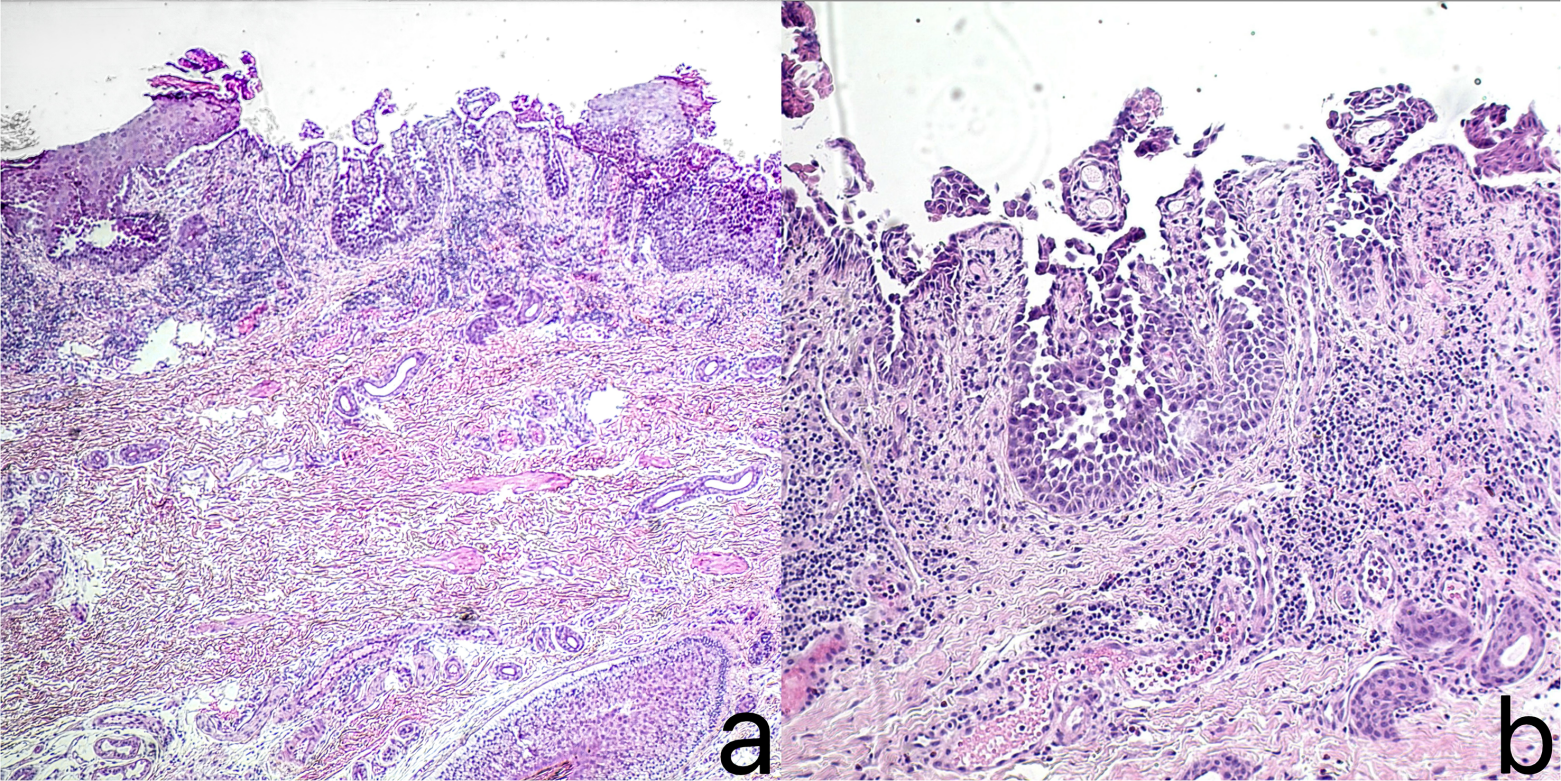
Histopathological examination of the left inguinal skin showed: local disruption of the epidermis, parakeratosis, acantholysis and blister formation above the basal layer, presence of dyskeratotic cells, and massive infiltration of lymphocytes-dominated inflammatory cells around the dermal blood vessels. (**(a)** H&E,×40; **(b)** H&E,×100).

A 38-year-old Chinese male patient presented with macerated hypertrophic erythema with surface erosion, breakdown, and exudation in the perianal area since 2 years ago, and similar rashes appeared in the groin bilaterally half a year ago (**Figures 1a–c**). His father had a similar condition. The patient had no other medical history. Pathological examination taken from the left groin showed localized epidermal breakdown, hyperkeratosis, loosening of the stratum spinosum above the basal layer, blister formation, visible dyskeratotic cells, and a large lymphocyte-dominated inflammatory cell infiltrate around the dermal blood vessels (**Figures 2a, b**). The diagnosis of Hailey-Hailey disease was made, and the patient had been treated with oral methylprednisolone tablets 20 mg/day, minocycline 200 mg/day, and topical mometasone furoate cream, tacrolimus, and fusidic acid with poor results. Now the patient urgently needed a new method of treatment, so we decided to give the patient upadacitinib 15mg/day treatment, before treatment to improve the complete blood count, liver and kidney function, coagulation function, hepatitis B virus test, hepatitis C virus test, human immunodeficiency virus test, tuberculosis gamma-interferon release test (IGRA), chest CT and so on, were no abnormality. The patient’s rash improved significantly at 3 weeks of treatment, and at 12 weeks of follow-up, the patient’s rash improved (**Figures 1d–f**), and no serious adverse reactions were observed.

## Discussion

Hailey-Hailey disease is a rare autosomal dominant disorder of echinodermolysis bullosa with clinical manifestations of recurrent blistering, vesiculation, and crusting in areas of skin folds. HHD is caused by a mutation in the ATP2C1 gene, which encodes for the human secretory pathway Ca2+/Mn2+-ATPase isoform 1 (hSPCA1) of the Golgi, a pump that provides the Golgi lumen to supply Ca2+ and Mn2+ and maintain their normal intracellular concentrations. Mutations in this gene result in reduced Golgi calcium stores and impaired calcium-dependent post-translational modification and protein sorting for transport, which in turn triggers the echinodermal loosening that characterizes the disease (6, 7). The prevalence of the disease is low, with some studies suggesting a prevalence of approximately 1 in 50,000, with onset occurring mostly after puberty (8). Approximately 70% of patients with Hailey-Hailey disease have a family history of the disease (9).The treatment of HHD includes traditional treatments such as oral and topical antibiotics, glucocorticoids, etc., surgical treatments, laser therapies (e.g. CO2 laser, Er: YAG laser ablation), radiofrequency surgeries, photodynamic therapy, electron-beam radiotherapy, and Botulinum toxin type A injections, etc. (10) as well as newer therapeutic options such as biologics, Janus kinase inhibitors, and other drugs, have been reported to be effective (11, 12). There are case reports showing successful treatment of refractory HHD after administration of upadacitinib (3).

The mechanism of HHD treatment with upadacitinib, a highly selective JAK1 inhibitor, can be analyzed from multiple dimensions. On the one hand, JAK1 plays a key role in the signaling of pro-inflammatory cytokines such as IL-4, IL-13, and IL-31 (13), which are abnormally overexpressed in HHD lesions and further exacerbate inflammatory responses and barrier damage by stimulating the release of chemokines (e.g., eotaxin-3) from keratinocytes (14). By inhibiting JAK1, upadacitinib can directly block the above inflammatory signaling pathway and alleviate the symptoms of skin erythema, itching and exudation (14). On the other hand, recent studies have shown that type 2 inflammatory factors (IL-4, IL-13) inhibit free calcium release and actin polymerization in keratinocytes, two processes that are central to maintaining intercellular adhesion (15). The selective inhibition of JAK1 by upadacitinib may indirectly restore calcium-dependent cellular junction stability and promote the repair of spiny loosening, which explains the rapid improvement of the patients’ lesions within a short period of time in the present study.

In recent years, in addition to JAK inhibitors, monoclonal antibody drugs have also demonstrated promising therapeutic potential for refractory Hailey-Hailey disease. Dupilumab, a human monoclonal antibody that blocks interleukin-4 and interleukin-13 receptors, has been successfully used in multiple clinical studies to treat refractory Hailey-Hailey disease. Relevant research indicates that all 11 patients treated with dupilumab achieved significant clinical improvement, with 64% achieving sustained disease remission lasting 5 to 24 months. No drug-related adverse reactions were observed. Its mechanism of action may involve suppressing Th2-mediated inflammatory responses (20–26), offering another important therapeutic option for this condition.

Here we conducted a systematic review of Janus kinase inhibitors treatment regimens in Hailey-Hailey disease and summarized five papers to explore the characteristics of their treatment regimens (**Table 1**). Literature reports showed a wide age range of patients, covering middle-aged to older age groups, ranging from 41 to 67 years old, with no significant difference in gender, with a male-to-female ratio of 3:2, and a disease duration ranging from 3 to more than 40 years. Most of the cases had a clear family genetic background, with four cases having a family history of the disease, two of which involved multiple family members across four generations (6 and 15, respectively), and only one case did not mention a family history, which reflects the genetic predisposition of the disease as an autosomal dominant disease, and the differences in the number of family members with the disease may be related to the rate of the disease-causing gene epitopes or the size of the family, which further confirmed the central role of the genetic factor in the pathogenesis of the disease. The central role of genetic factors in the pathogenesis of the disease is further confirmed.

**Table 1:** Summary of family history and treatment with Janus kinase inhibitors in Hailey-Hailey disease.

Author	Age/Gender	Duration of Hailey-Hailey	Family history	Drug name	Dosage	Treatment duration	Side effects	Therapeutic efficacy
Murphy, L, et al. (3)	65/woman	more than forty years	The father has a similar disease.	upadacitinib	15mg once daily	4 months	Not mentioned	After 4 weeks of treatment, the active lesions healed, leaving only a small amount of post-inflammatory erythema; the lesions continued to improve at 12 weeks; and at the 16-week follow-up, the lesions were cleared with no recurrence.
Gunyon, M, et al. (16)	60/woman	more than forty years	Six family members across four generations have the disease.	abrocitinib	100mg once daily	Not mentioned	mild, tolerable nausea, and constipation	The patient achieved complete resolution of skin lesions within 2 weeks of abrocitinib treatment, with recurrence after 2 weeks of drug discontinuation but regained complete control upon resumption
Li, Y, et al. (17)	41/man	twelve years	Fifteen family members across four generations have developed similar symptoms.	abrocitinib	100mg once daily	4 months	Not mentioned	After 4 weeks of treatment, the clinical symptoms improved significantly. With continued treatment for 4 months, no new rashes appeared.
Bao, H, et al. (18)	67/man	three years	N/A	tofacitinib	5mg BID	6 months	No side effects	significant improvements after two weeks: DLQI dropped by 15 points, PGA decreased from 3 to 1, BSA improved over 90%.Maintenance treatment for 6 months led to sustained symptom remission without recurrence.
Cui, Y, et al. (19)	48/man	more than twenty years	his mother, brother and sister also had a similar condition	upadacitinib	15mg once daily	6 month	No side effects	marked improvement of HHD within 1 month of upadacitinib treatment.during 6-month follow-up, no HHD flare-ups

In terms of treatment, various types of Janus kinase inhibitors have shown significant efficacy. Among the specific drugs, Janus kinase inhibitors such as upadacitinib, abcixitinib, tofacitinib, etc., used alone or in combination with topical drugs, can effectively improve the symptoms of erythema, blisters, and vesicles, and in some cases can achieve rapid remission or long-term stabilization, which provides an important option for patients who have poor results from traditional treatments, for example, after the use of upadacitinib in a 65-year-old female patient as reported by Murphy, L et al., the active lesions in 4 weeks largely healed and completely resolved without recurrence at 16-week follow-up (3); Gunyon, M et al. reported complete resolution of skin lesions within 2 weeks of abcixitinib treatment in the case of a 60-year-old female, suggesting that highly selective JAK1 inhibitors may offer a highly effective therapeutic option for HHD (16). In combination therapy, Janus kinase inhibitors in combination with topical glucocorticoids, antimicrobials or zinc oxide oil were effective in improving skin lesions and had a good safety profile, with no serious adverse effects at 6-month follow-up, suggesting that the combination of systemic and topical drugs may enhance the efficacy of therapy. For example, Li, Y et al. reported that a 41-year-old male patient was treated with abcixitinib in combination with topical zinc oxide oil, which resulted in a significant improvement of clinical symptoms after 4 weeks and continued treatment for 4 months without new rash (17); Cui, Y et al. reported that a 48-year-old male patient treated with ubacitinib in combination with topical mometasone furoate and fusidic acid cream for 1 month was switched to tofacitinib in combination with the same topical agent, and had stable disease without recurrence and no adverse effects during the 6-month follow-up period (19).

Given the chronic nature of HHD and the treatment goal of long-term remission, combined with existing clinical evidence and data from this study, we propose the following: For patients with mild to moderate HHD presenting localized skin lesions, low recurrence frequency, and minimal impact on quality of life, JAK inhibitors may serve as a short-term induction therapy regimen. A 12-week treatment course should be selected, with significant symptom improvement as the primary endpoint, thereby avoiding prolonged systemic drug exposure. For patients with severe refractory HHD presenting extensive, recurrent lesions unresponsive to conventional therapies, long-term maintenance therapy is required. Clinical cases have validated the efficacy of this strategy (3, 16–19). It is crucial to emphasize that treatment duration should be individualized, dynamically adjusted based on lesion clearance, recurrence frequency, and safety monitoring results. For patients demonstrating good treatment response, gradual dose reduction may be considered to minimize potential medication risks while maintaining therapeutic efficacy.

## Conclusion

Hailey-Hailey disease is characterized by typical clinical features, high difficulty in traditional treatment, and limited evidence-based protocols. In this case, after 12 weeks of treatment with upadacitinib, the patient axillary and inguinal lesions and itchy symptoms improved significantly, and laboratory tests were normal with no adverse effects, demonstrating a positive therapeutic potential. This provides new ideas and references for clinicians to deal with similar patients who have been poorly treated with conventional therapies. Given that there are few studies on Janus kinase inhibitors in the treatment of Hailey-Hailey disease and most of them are case reports, multicenter, large-sample, and long-term follow-up clinical trials are needed to further validate the long-term efficacy and safety of Janus kinase inhibitors such as upadacitinib, in order to optimize the therapeutic strategies and to provide more solid evidence for the clinical management of this difficult-to-treat disease.

## Data Availability

The original contributions presented in the study are included in the article/supplementary material. Further inquiries can be directed to the corresponding author.
